# Impact of the Coronavirus Disease 2019 Pandemic on the Patterns and Characteristics of Plastic Surgery Practice: A Retrospective Comparative Study of Before and During the Pandemic

**DOI:** 10.7759/cureus.29722

**Published:** 2022-09-28

**Authors:** Hatan Mortada, Ziyad H Alawaji, Rema A Aldihan, Lamia A Alkuwaiz, Sultan F Alshaalan, Abdullah E Kattan

**Affiliations:** 1 Department of Plastic Surgery & Burn Unit, King Saud Medical City, Riyadh, SAU; 2 Division of Plastic Surgery, Department of Surgery, King Saud University Medical City, King Saud University, Riyadh, SAU; 3 College of Medicine, Qassim University, Buraydah, SAU; 4 College of Medicine, King Saud University, Riyadh, SAU; 5 Division of Plastic Surgery, Department of Surgery, College of Medicine, King Saud University, Riyadh, SAU

**Keywords:** practice pattern, retrospective study, pandemic, plastic surgery, covid-19

## Abstract

Background

Plastic surgery practices have changed drastically during the coronavirus disease 2019 (COVID-19) pandemic, as most non-elective surgeries were deferred owing to the high presumed risk of infection transmission. Therefore, this study aimed to assess the impact of the COVID-19 pandemic on the patterns and characteristics of plastic surgery in an academic medical city.

Methods

This retrospective comparative study was conducted at King Saud University Medical City, Riyadh, Saudi Arabia. We reviewed data from patient medical records during the pandemic period (from March 2, 2020, to December 31, 2020) and the corresponding pre-pandemic period, defined as the same timeframe in the preceding year (from March 2, 2019, to December 31, 2019).

Results

The total number of admitted patients in the pre-pandemic period was 479 and in the during-pandemic period was 254, indicating a 46.97% reduction in admission frequency. The median length of hospital stay was significantly shorter in 2020 than in 2019 (1.62 ± 1.74 days versus 2.13 ± 4.18, respectively, p = 0.011). The during-pandemic period was characterized by significantly higher frequencies of urgent procedures (9.8% versus 5.4% in 2020 and 2019, respectively, p = 0.025) and lower frequencies of elective procedures (90.2% vs. 94.4% in 2020 and 2019, respectively, p = 0.035) than the pre-pandemic period.

Conclusion

The number of plastic surgeries performed has decreased since the onset of the pandemic. However, the impact of the pandemic on plastic surgery practices remains unclear. Further studies are needed to determine the effects of this pandemic on patient outcomes.

## Introduction

Coronavirus disease 2019 (COVID-19) is a highly contagious disease that first appeared in Wuhan, China, in December 2019 [1]. In March 2020, COVID-19 was officially declared a global pandemic [1]. This pandemic has since had an extremely detrimental impact on the world. Governments have enforced major lockdowns, travel restrictions, and border shutdowns, which have combined to cause major economic crises [2]. Furthermore, COVID-19 is a significant obstacle to all healthcare systems worldwide. The surgical field is no exception, as surgery has been reported to worsen the disease progression of COVID-19 [3].

Plastic surgery practices have changed drastically during the pandemic, as many non-elective surgeries were deferred owing to the high presumed risk of infection transmission. The International Society of Aesthetic Plastic Surgery recommends minimizing or postponing aesthetic procedures as much as possible [4]. Since the beginning of the pandemic, several studies reporting the impact of COVID-19 on plastic surgery have been published. These studies all showed a significant decline in the number of surgeries in both academic and private practice and, to a greater extent, in private practice [5-8]. As the number of cases began to decline, restrictions on elective surgeries began to decrease. Many institutions, including ours, require preoperative polymerase chain reaction (PCR) test results to ensure the safety of patients and healthcare workers. Although this can minimize the risk of infection transmission, false-negative test results remain a possibility [9]. A survey-based study by Teitelbaum et al. demonstrated that plastic surgeries could be safely conducted even during a significant pandemic surge [10]. Furthermore, Brown et al. investigated 350 elective surgical procedures and indicated a minimal risk of COVID-19 transmission with appropriate screening and safety precautions [11]. This study aimed to assess the impact of the COVID-19 pandemic on the patterns and characteristics of plastic surgery practices in King Saud University Medical City, Riyadh, Saudi Arabia.

## Materials and methods

This study was a retrospective, comparative study performed at a single center. We enrolled all consecutive plastic surgical patients who underwent surgical intervention from the start of the COVID-19 pandemic in Saudi Arabia.

Patient records during the pandemic period (from March 2, 2020, to December 31, 2020) and the corresponding pre-pandemic period in the same timeframe in the preceding year (from March 2, 2019, to December 31, 2019) were reviewed. This study was approved by the Institutional Review Board and Research Ethics Committee of King Saud University, Riyadh, Saudi Arabia (IRB No. 0092/22). Data were retrospectively collected and uploaded to a Microsoft Excel sheet (Microsoft Corporation, Redmond, WA) from the hospital's electronic medical records and patient charts. We identified the demographics (including age and sex), comorbidities, smoking status, admission and discharge dates, diagnosis, diagnosis category, the surgical procedure performed, type of anesthesia, length of hospital stay, priority category, complications, and mortality of the patients. Patient triaging priority was defined by the use of the following four categories: Category 1 (immediate - within minutes), Category 2 (urgent - within hours), Category 3 (expected - within days), and elective (planned).

To ensure the reliability of the analysis, we compared the frequencies and characteristics of admissions during the pandemic period (from March 2, 2020, to December 31, 2020) and the corresponding pre-pandemic period in the same timeframe in the preceding year (from March 2, 2019, to December 31, 2019). Additionally, we analyzed the percentage change in admissions between the lockdown period (from March 25, 2020, to June 21, 2020) and the same period in 2019. Descriptive data were used to express categorical variables (frequencies and percentages) and numerical variables (means and standard deviations (SD)). Statistical differences between the pre and during-pandemic periods were assessed using the Mann-Whitney U test for continuous data (e.g., length of hospital stay) and the chi-squared test for categorical data. A p-value of < 0.05 was considered statistically significant. The collected data were coded, entered, and analyzed using the Statistical Package for the Social Sciences (IBM Corp. Released 2019. IBM SPSS Statistics for Windows, version 26.0. Armonk, NY: IBM Corp). Summary and display of the data were performed using descriptive methods.

## Results

Frequency of admissions

**Figure 1:**
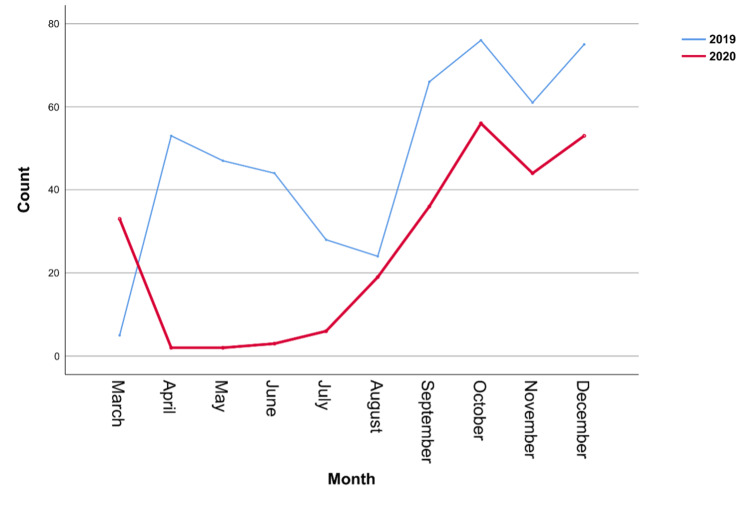
The frequencies of patients admitted for plastic surgery across different months in the pre and during-pandemic periods

The total number of admitted patients was 479 in the pre-pandemic period and 254 in the during-pandemic period, representing a 46.97% reduction in the frequency of admissions. The number of patients admitted for plastic surgery was generally lower during the pandemic period from April to December 2020 than in the same period in 2019 (Figure 1).

Focusing on the analysis of the lockdown period in 2020 (March 25 to June 21), we reported seven admissions, which was lower than that reported in the same period in 2019 (n=126). This represented a 94.44% reduction in the number of admissions between the two periods.

Demographic characteristics of patients

There was no difference in patients’ sex distribution between the pre and during-pandemic periods. However, significantly higher proportions of patients aged zero to five years were admitted to our institution during the pandemic period than in the same timeframe in 2019 (15.4% versus 6.9% in 2020 and 2019, respectively, p < 0.0001). Additionally, the frequency of admissions of pediatric patients (aged <18 years) was significantly higher in the during-pandemic period than in the pre-pandemic period (25.2% vs. 16.3%, p = 0.004). Conversely, admissions were significantly lower in 2020 than in 2019 among patients aged 40-60 years (24.4% vs. 34.7%, p = 0.004) and among smokers (3.5% vs. 11.3%, respectively, p < 0.001, Table 1).

**Table 1:** Demographic characteristics of the patients

Parameter	Category	Pre-pandemic (N=479)	During-pandemic (N=254)	p
Sex	Male	199 (41.5)	108 (42.5)	0.799
	Female	280 (58.5)	146 (57.5)	
Age	0–5 y	33 (6.9)	39 (15.4)	< 0.0001
	5–8 y	45 (9.4)	25 (9.8)	0.844
	18–40 y	211 (44.1)	113 (44.5)	0.910
	40–60 y	166 (34.7)	62 (24.4)	0.004
	> 60 y	24 (5.0)	15 (5.9)	0.607
Pediatrics (<18 years)	Yes	78 (16.3)	64 (25.2)	0.004
Smoking status	Smoker	54 (11.3)	9 (3.5)	< 0.0001
	Nonsmoker	342 (71.4)	245 (96.5)	< 0.0001
	Not mentioned	83 (17.3)	0 (0.0)	< 0.0001

**Figure 2:**
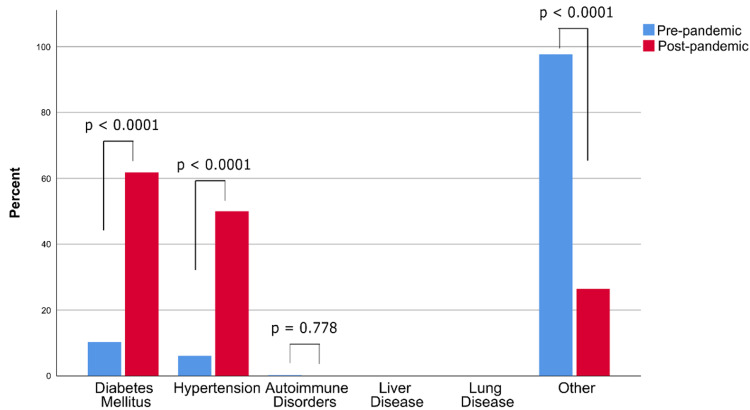
The frequencies of comorbidities among patients admitted for plastic surgeries during the pre and during-pandemic periods

Regarding comorbid conditions at presentation, there were significant differences in the proportions of patients with diabetes mellitus (61.8% in 2020 compared to 10.3% in 2019, p < 0.0001), hypertension (50.0% vs 6.1%, p < 0.0001), and other comorbid conditions (26.5% vs 97.7%, p < 0.0001, Figure 2).

Characteristics of admissions and surgeries

The median length of hospital stay was significantly shorter in 2020 than in 2019 (1.62 ± 1.74 days versus 2.13 ± 4.18, respectively, p = 0.011). The during-pandemic period was characterized by significantly higher frequencies of urgent procedures (9.8% versus 5.4% in 2020 and 2019, respectively, p = 0.025), and lower frequencies of elective procedures (90.2% vs. 94.4% in 2020 and 2019, respectively, p = 0.035). Furthermore, we observed significant differences in the frequencies of distinct surgeries in 2020 compared to 2019, where the proportions of hand and upper extremity surgeries were significantly higher (59.1% vs. 49.3%, p = 0.012), and craniofacial surgeries were significantly lower (7.1% vs. 15.0%, p = 0.002). Regarding the employed anesthesia, the proportions of surgeries requiring general anesthesia were significantly higher in the during-pandemic period than in the pre-pandemic period (68.9%% versus 61.4%%, p = 0.044, Table 2).

**Table 2:** Characteristics of surgeries performed at our institution in the pre and during-pandemic periods

Parameter	Category	Pre-pandemic (N=479)	During-pandemic (N=254)	p
Urgency of surgery	Elective (planned admission for surgery)	449 (93.7)	231 (90.9)	0.165
	Emergency (unplanned admission)	30 (6.3)	23 (9.1)	
Classification of surgical	Category- 1	1 (0.2)	0 (0.0)	0.466
	Category - 2	26 (5.4)	25 (9.8)	0.025
	Elective	452 (94.4)	229 (90.2)	0.035
Diagnosis	Hand and upper extremity	236 (49.3)	150 (59.1)	0.012
	Craniofacial surgeries	72 (15.0)	18 (7.1)	0.002
	Pediatric plastic surgery	25 (5.2)	18 (7.1)	0.306
	Burn surgeries	42 (8.8)	19 (7.5)	0.578
	Aesthetic surgeries	46 (9.6)	22 (8.7)	0.692
	Breast reconstruction and microsurgery	58 (12.1)	27 (10.6)	0.552
Anesthesia	General anesthesia	294 (61.4)	175 (68.9)	0.044
	Spinal anesthesia	1 (0.2)	0 (0.0)	0.466
	Nerve block	4 (0.8)	1 (0.4)	0.490
	Local anesthesia	180 (37.6)	78 (30.7)	0.064

Carpal tunnel syndrome (CTS) was the most common reason for admission in both the pre-pandemic (9.2%) and during-pandemic (10.2%, Table 3) periods.

**Table 3:** The most common reasons for patient admission in the pandemic periods

Pre-pandemic (N=479)		During-pandemic (N=254)	
Condition	Frequency (%)	Condition	Frequency (%)
Carpal tunnel syndrome	44 (9.2)	Carpal tunnel syndrome	26 (10.2)
Scar deformity	43 (9.0)	Skin redundancy	22 (8.7)
Skin redundancy	29 (6.1)	Trigger finger	20 (7.9)
Trigger finger	27 (5.6)	Scar deformity	19 (7.5)
Ganglion cyst	19 (4.0)	Cleft palate	17 (6.7)
Lipoma	17 (3.5)	Hand injury	13 (5.1)
Metacarpal fracture	17 (3.5)	Breast hypertrophy	12 (4.7)
Contracture deformity	14 (2.9)	Lipoma	12 (4.7)
Mass	14 (2.9)	Ganglion cyst	11 (4.3)
Wound defect	12 (2.5)	Flexor tendon injury	10 (3.9)

Excision procedures and CTS release were the most commonly performed surgeries in the pre-pandemic (22.8% and 7.1%, respectively) and during-pandemic periods (16.9% and 9.4%, respectively; Table 4).

**Table 4:** The most common surgeries performed in the pre and during-pandemic periods CTS: carpal tunnel syndrome

Pre-pandemic (N=479)		During-pandemic (N=254)	
Surgery	Frequency (%)	Surgery	Frequency (%)
Excision	109 (22.8)	Excision	43 (16.9)
CTS release	34 (7.1)	CTS release	24 (9.4)
Release	33 (6.9)	Exploration	19 (7.5)
Exploration	23 (4.8)	Cleft palate repair	16 (6.3)
Abdominoplasty	20 (4.2)	Breast reduction	15 (5.9)
Trigger finger release	20 (4.2)	Release	14 (5.5)
Debridement	18 (3.8)	Abdominoplasty	12 (4.7)
Breast reduction	15 (3.1)	Trigger finger release	11 (4.3)
Scar revision	13 (2.7)	Liposuction	6 (2.4)
Skin graft	11 (2.3)	Skin graft	6 (2.4)

Surgical outcomes

No mortality was reported in our institution during the study period. The reported complications included pneumonia, acute respiratory distress syndrome, and neuropraxia (n=1 each) in the pre-pandemic period, with no significant differences compared with those in 2020. Additionally, deep venous thrombosis occurred in one patient in the pre-pandemic period and one patient in the during-pandemic period. Lastly, neuropraxia occurred in one patient in the pre-pandemic period.

## Discussion

During the pandemic, a significant reduction in the admission rate was observed. The frequency of admissions to the plastic surgery department was reduced by approximately 47%. The reduction was particularly remarkable during the lockdown period from March 25 to June 21, 2020, with a reduction rate of 94.44%. This finding is consistent with other similar studies conducted in Iran and southern India, where a significant decline in the number of plastic surgeries conducted after the pandemic was reported [8,12]. The reduction in the admission rate is thought to be partly due to the hospital's policy of prioritizing urgent and emergent cases and partly because of patients' fear of contracting COVID-19 infection in the hospital. This reduction is beneficial in terms of increasing the hospital capacity for COVID-19 patients, minimizing the cross-infection of COVID-19, and preserving PPE for health care workers [13]. However, postponing this huge number of surgeries could cause a significant setback in the healthcare system. Indeed, Wiseman et al. previously highlighted the massive impact of COVID-19 on surgical waitlists [14].

Our study found that the number of pediatric patients admitted was significantly higher in the during-pandemic period than in the pre-pandemic period. There was an 8.5% increase in the admission rate for the zero to five years age group and an 8.9% increase in the pediatric age group (aged <18 years). In contrast, there was a 10.3% decrease in the adult age group admission rate (40-60 years) (Table 1). Furthermore, we noticed a significant increase in the admission rates among patients with comorbidities, diabetes, and hypertension by 51.5% and 43.9%, respectively (Figure 2). 'Significant Comorbidities' has been a way to define major surgeries preoperatively [15]. This may be a reason for the increase in the number of admissions of patients with significant comorbidities such as diabetes and hypertension. We believe that these changes can be attributed to the restrictions implemented by the hospital in prioritizing urgent and emergent cases admitted for surgery. As for the elective procedures mentioned in this article during the pandemic, most of these patients were electively operated on after the lockdown was lifted. Prior to surgery, all patients had a negative COVID-19 swap.

Moreover, the hospital stay was significantly shortened compared to the pre-pandemic period. This was observed in many centers worldwide, as many centers have been reported to have implemented enhanced recovery programs [13]. Furthermore, early discharge was proven to be both safe and feasible. Sica et al. described that coping with these protocols was facilitated by the high awareness of infectious risk among patients [16]. A significant reduction in the rate of craniofacial surgeries was also observed in this study. This finding is consistent with that of a similar study that reported a 52% reduction in craniofacial surgeries [12]. This might be attributed to the riskiness of the operative area, as infected individuals tend to have the highest viral load in the pharynx and upper aerodigestive tract [17]. Our study reported a higher incidence of urgent procedures after the pandemic. Conversely, a similar study conducted in the US reported fewer emergency cases admitted following plastic surgery than in 2019 [18].

In addition, during the study period, no mortality was documented in the plastic surgery department. After surgery in the plastic surgery division, none of the included patients contracted COVID-19. Four patients experienced at least one complication during their hospital stays in the pre-pandemic period (a single case of pneumonia, acute respiratory distress syndrome, neuropraxia, and deep vein thrombosis (DVT)). Regardless of the increase in admission of patients with significant comorbidities, which has been documented as being associated with a higher risk of poor outcomes [19,20], there were no significant postoperative complications documented during the pandemic period except for a single reported case of DVT.

Limitations

The study has several limitations. First, data were collected using a retrospective design and from a single-center experience. Therefore, our results may not be representative of all hospitals. Furthermore, with the ongoing pandemic, the situation is expected to change over time. Additionally, the data did not include the effects of postponing surgeries for patients on the waiting list, which is considered an essential factor in assessing the outcomes of the pandemic.

## Conclusions

This study provides a retrospective comparison of the practices of the plastic surgery department in a university hospital before and after the COVID-19 pandemic. There has been a notable decrease in the number of surgeries performed since the onset of the pandemic in March 2020. As a historical record and for determining the effects of the pandemic on plastic surgery practice, the information presented in this article is crucial. The impact of the pandemic on plastic surgery practices remains unclear. More studies are needed to determine the effect of the COVID-19 situation on patient outcomes, as it is an unprecedented situation in modern history. In addition, the pandemic's financial impact must be considered.

## References

[1] Wu F, Zhao S, Yu B (2020). A new coronavirus associated with human respiratory disease in China. Nature.

[2] Nicola M, Alsafi Z, Sohrabi C (2020). The socio-economic implications of the coronavirus pandemic (COVID-19): a review. Int J Surg.

[3] Lei S, Jiang F, Su W (2020). Clinical characteristics and outcomes of patients undergoing surgeries during the incubation period of COVID-19 infection. EClinicalMedicine.

[4] (2022). ISAPS. COVID- 19: Recommendations for management of elective surgical procedures in aesthetic surgery. https://www.isaps.org/covid-19/covid-19-recommendations-for-management-of-elective-surgical-procedures-in-aesthetic-surgery/.

[5] Sarac BA, Schoenbrunner AR, Wilson SC, Chiu ES, Janis JE (2022). The impact of COVID-19-based suspension of surgeries on plastic surgery practices: a survey of ACAPS members. Plast Reconstr Surg Glob Open.

[6] Al Bayati MJ, Samaha GJ, Wo LM, Wolfe EM, Samaha MJ, Kassira WM, Lessard AS (2021). Operational and financial impact of COVID-19: a survey of plastic surgeons in Miami. Plast Reconstr Surg Glob Open.

[7] Joji N, Nugent N, Vadodaria S, Sankar TK (2021). Impact of COVID-19 on aesthetic plastic surgery practice in the United Kingdom. J Plast Reconstr Aesthet Surg.

[8] Chellamuthu A, Kumar JS, Ramesh BA (2021). Impact of COVID -19 pandemic on plastic surgery practices in a tertiary care set up in southern India. Niger J Clin Pract.

[9] West CP, Montori VM, Sampathkumar P (2020). COVID-19 testing: the threat of false-negative results. Mayo Clin Proc.

[10] Teitelbaum S, Diaz J, Singer R (2021). Can outpatient plastic surgery be done safely during a COVID-19 surge? Results of a July 2020 Los Angeles survey and literature review. Aesthet Surg J.

[11] Brown M, Eardley S, Ahmad J (2021). The safe resumption of elective plastic surgery in accredited ambulatory surgery facilities during the COVID-19 pandemic. Aesthet Surg J.

[12] Kalantar-Hormozi A, Habibzadeh Z, Yavari M (2021). Impact of COVID-19 pandemic on plastic surgery activities and residency programs in a tertiary referral centre in Iran. Eur J Plast Surg.

[13] Soltany A, Hamouda M, Ghzawi A, Sharaqi A, Negida A, Soliman S, Benmelouka AY (2020). A scoping review of the impact of COVID-19 pandemic on surgical practice. Ann Med Surg (Lond).

[14] Wiseman SM, Crump RT, Sutherland JM (2020). Surgical wait list management in Canada during a pandemic: many challenges ahead. Can J Surg.

[15] Martin D, Mantziari S, Demartines N, Hübner M (2020). Defining major surgery: a Delphi consensus among European Surgical Association (ESA) members. World J Surg.

[16] Sica GS, Campanelli M, Bellato V, Monteleone G (2020). Gastrointestinal cancer surgery and enhanced recovery after surgery (ERAS) during COVID-19 outbreak. Langenbecks Arch Surg.

[17] Andrews BT, Garg R, Przylecki W, Habal M (2020). COVID-19 pandemic and its impact on craniofacial surgery. J Craniofac Surg.

[18] Paiva M, Rao V, Spake CS (2020). The impact of the COVID-19 pandemic on plastic surgery consultations in the emergency department. Plast Reconstr Surg Glob Open.

[19] Zhou F, Yu T, Du R (2020). Clinical course and risk factors for mortality of adult inpatients with COVID-19 in Wuhan, China: a retrospective cohort study. Lancet.

[20] Wu C, Chen X, Cai Y (2020). Risk factors associated with acute respiratory distress syndrome and death in patients with coronavirus disease 2019 pneumonia in Wuhan, China. JAMA Intern Med.

